# Memory and mental time travel in humans and social robots

**DOI:** 10.1098/rstb.2018.0025

**Published:** 2019-03-11

**Authors:** Tony J. Prescott, Daniel Camilleri, Uriel Martinez-Hernandez, Andreas Damianou, Neil D. Lawrence

**Affiliations:** 1 Department of Computer Science and Sheffield Robotics, University of Sheffield, Sheffield, UK; 2 Department of Electronic and Electrical Engineering, University of Bath, Bath, BA2 7AY, UK; 3 Amazon, I Station Square Road, Cambridge, CB1 2GA, UK

**Keywords:** mental time travel, autobiographical memory, latent variable space, Gaussian process, symbol grounding

## Abstract

From neuroscience, brain imaging and the psychology of memory, we are beginning to assemble an integrated theory of the brain subsystems and pathways that allow the compression, storage and reconstruction of memories for past events and their use in contextualizing the present and reasoning about the future—mental time travel (MTT). Using computational models, embedded in humanoid robots, we are seeking to test the sufficiency of this theoretical account and to evaluate the usefulness of brain-inspired memory systems for social robots. In this contribution, we describe the use of machine learning techniques—Gaussian process latent variable models—to build a multimodal memory system for the iCub humanoid robot and summarize results of the deployment of this system for human–robot interaction. We also outline the further steps required to create a more complete robotic implementation of human-like autobiographical memory and MTT. We propose that generative memory models, such as those that form the core of our robot memory system, can provide a solution to the symbol grounding problem in embodied artificial intelligence.

This article is part of the theme issue ‘From social brains to social robots: applying neurocognitive insights to human–robot interaction’.

## 1. Introduction

Mental time travel (MTT) describes the capacity to project the mind back in time to recover memories of past events, and forward in time to imagine possible future events [1]. Neuropsychological studies of amnesia, together with functional brain imaging studies, have demonstrated that MTT, to either the past or future, involves a similar set of brain subsystems and pathways [2]. Given the starting point of a cue corresponding to a past event, this system can fill out the wider context and retrieve what happened next; or, in the case of the imagined future, the same system can construct how a scenario could unfold in the light of past experience.

To disconnect from the present, in order to contemplate the past or future, allows the mind to reflect on its own existence in space and time. MTT, and the memory systems underlying it, thus make an important contribution to human self-awareness [1] and to the temporally extended sense of self [3]. These same systems also support our capacity for episodic/autobiographical memory—the ability to recall and relive episodes from our own life history [4]. Autobiographical memory, in turn, provides the material from which we construct a life story that we then use to communicate to others about who we believe we are [5].

The research described in this article follows an ‘understanding through building’ approach that seeks to advance the theoretical understanding of the mind and brain by testing computational models in embodied systems (robots) [6,7]. This approach thus combines the analysis of biological systems with the synthesis of models that embed key biological principles. Models can be built at different levels of abstraction, allowing, for instance, models of spiking neural networks of specific neural circuits to be compared with functional models of brain systems specified in more algorithmic terms. A physical model in the form of a robot can stand as an existence proof that the theoretical model that this embodies can generate the animal or human behaviour we wish to capture; in other words, it provides evidence for the sufficiency of the model and theory [8].

An added benefit of this approach is that an embodied model can also act as a prototype for a useful technology—in our case as a demonstration of the possibility of autobiographical memory and MTT for robots. The current generation of intelligent devices—from phones, to personal computers, to robots—have an increasingly impressive capacity for gathering and storing information in a range of useful modalities (sound, vision, even touch and smell); however, they remain surprisingly poor at retrieving relevant information when it is needed [9]. Indeed, the capacity to archive more and more information, facilitated by the availability of cheap and compact memory hardware, is pushing us towards a situation where we can record everything—so-called life-logging in the case of wearable computers [10]—but where, in reality, we will never have the time to filter, retrieve or evaluate even a small fraction of the information that has been stored. In the area of robotics, we are also beginning to see the development of social robots that can interact with people on a daily basis, assisting in domestic tasks, providing support for people with disabilities, even providing some capacity for social companionship [11–14]. Nevertheless, despite having the possibility to store virtually everything that happens to them, such machines are poor at knowing what aspects of their own history are relevant to making decisions, performing actions or engaging with people in the here and now. This is partly the well-known ‘frame problem’ in artificial intelligence (AI), and the solution, at least in part, to this problem of knowing what is important, is to be able to relate past experience to identity and purpose [15]. The ability to recall past interactions with others, to share memories and to swap ideas about the future is also central to human relationships; that today's robots are ‘marooned in the present’ therefore limits their capacity to be useful or engaging as social partners.

Based on these considerations, we have sought to create a biomimetic memory system that can help address the challenge of improving the social capabilities of robots. We next outline our general approach to creating this system by describing its genesis from psychology, brain theory and machine learning. We then summarize how we have implemented a form of synthetic multimodal memory for the iCub humanoid robot and provide results from its deployment in human–robot interaction. We conclude by considering how such a system could be extended towards a more complete model of human-like autobiographical memory and MTT, and how that might be used within the control systems of next-generation social robots.

## 2. From brain theory to synthetic memory

Autobiographical memory and MTT are holistic capabilities of the human mind/brain that depend on multiple neural substrates, bodily and sensory systems, and their interaction within different environmental and social contexts. In this section, we briefly summarize a theoretical framework for understanding human memory and outline some of its modes of operation, we then introduce a general approach to memory modelling that will allow us to emulate aspects of this framework within a control system for the iCub humanoid robot.

### (a) A framework for understanding human memory

**Figure 1.**
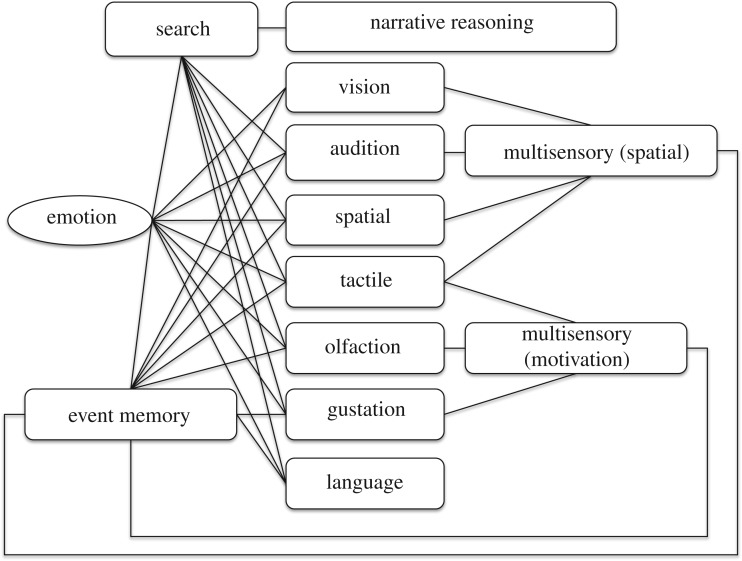
A model of human event memory proposed by Rubin [4,16] adapted from Rubin [4]. See text for further details.

Figure 1 illustrates a theoretical framework proposed by Rubin [4,16] and grounded on data from experimental psychology concerning the properties and capabilities of human autobiographical memory, and from neuropsychology and neuroimaging concerning its likely neural substrates. These findings include differences in recollection and ratings of memory vividness [17–19], under varying conditions of visual and auditory imagery and emotional significance, and evidence of the localization of different aspects of memory processing in multiple brain regions during recollection episodes, as indicated by studies of people with amnesia [20] and by patterns in fMRI activity [21,22]. Collectively, these findings indicate that a broad and distributed brain network underlies our ability to store and recall life experience. More specifically, this model proposes that autobiographical memory is generated by the interaction of (i) multiple within-modality stimulus-encoding subsystems; (ii) multisensory integration subsystems including those that integrate (a) visual, tactile and auditory inputs to provide a unified representation of local space, and (b) visual, olfactory, gustatory, tactile and emotional signals to encode reward value; (iii) a narrative reasoning subsystem that can prime search; and (iv) an event memory subsystem that binds multisensory information to encode traces of specific experienced events. The different elements of this system can be mapped to distinct brain areas, the sensory areas to the relevant cortical (and subcortical) sensory regions, the multisensory areas to some of the integrative capabilities of the sensory [23] and orbitofrontal [24] cortices, emotional areas to the limbic system and event memory to the extended hippocampal system (e.g. [25,26] and below). In the model, multiple subsystems are involved in encoding and retrieving memories, with the event memory subsystem serving specifically to bind together the multiple elements, contemporaneous or sequential, of an event.

Rubin's framework was primarily conceived of as a model of autobiographical memory; however, the same network could also support imagination if primed with a cue corresponding to an imagined future event rather than an actual past event. This proposal is supported by evidence that the ‘default network’, a system of interlinked areas of the temporal, frontal, parietal, cingulate and retrosplenial cortices, shows increased activity when people are awake but, at rest, has been found to be active during both memory and imagining (see [2] for review and [27] for a detailed discussion of this close relationship between episodic memory and MTT).

Studies of human autobiographical memory further indicate that while memories are recovered via the loop through the hippocampal system, the outputs of that system generate activity within sensory, parietal and prefrontal areas that reconstruct the recollected event [28]. As described by Daselaar *et al*. [21], recall follows a clearly defined temporal trajectory—early activity in the hippocampus, and in related structures such as retrosplenial cortex, is thought to correspond with accessing the memory trace, while later activity in the visual, left prefrontal and parietal cortices appears to be involved in its reconstruction. This interpretation is supported by evidence that the reported ‘vividness’ of recalled memories is correlated with the strength of activity in visual and prefrontal areas. Activity in the amygdala, correlated with emotional intensity of the recalled event, and occurring during the early access phase, suggests a role for emotion in cueing recall [4].

### (b) Latent variable models of memory

It is a widely held tenet of associative and connectionist theories of memory that we can think of the instantaneous pattern of neural firing across a distributed neural network as a point in high-dimensional space, and of the temporal dynamics of activity changes in such a network as a trajectory through such a space (e.g. [29,30]). A second starting point for the current model is therefore the hypothesis that the different subsystems that comprise figure 1 can be considered as *latent variable* (LV) spaces whose dimensions encode salient characteristics of the physical and social world. The dimensions of each variable space are latent because they are inferred by the brain rather than given in the sensory data; this makes the nature of memory encoding modality-specific (different LV representations for different stimulus types) while relying on universal principles for encoding/decoding. To efficiently encode rich data streams, a key property of these subsystems must be the capacity to reduce the bandwidth of incoming signals; however, memory must also be a *generative* process if it is to rekindle patterns of brain activity that are similar to those induced by the original event. In other words, the different memory subsystems must support both *pattern compression*—the encoding of an event in a compact and efficient way, and *pattern generation*—the reconstruction of the event given a cue. Pattern generation must allow both *pattern completion*—the filling out of a memory based on a fragment or a partial cue, and *pattern separation*—the ability to recall, as distinct patterns, events that share some of the same sensory properties. More specifically, and as described next, in implementing a version of the model shown in figure 1, we will view the operation of the sensory and multisensory memory systems as being analogous to learning processes that discover useful low-dimensional LV descriptions of high-dimensional data, and that operate bi-directionally both to encode high-dimensional stimulus patterns and to reconstruct such patterns from their low-dimensional description.

Many computational models have been proposed that capture interesting properties of human memory at different levels of abstraction and biological detail. Our interest is in matching the functional properties of human autobiographical memory and MTT and in creating a system that can operate in real time during human–robot interaction. We have therefore selected a modelling approach that is computationally efficient that can support the requirements for pattern compression/reconstruction discussed above, and that is relatively easy to interrogate and understand. Specifically, we employ an abstraction of biological memory processes that directly implements the notion of LV space called *Gaussian process latent variable models* (GP-LVMs) [31]. GP-LVMs are probabilistic, non-parametric equivalents of neural networks that have many useful properties including the ability to discover highly compressed LV spaces and to act as content-addressable memories and generative models. Statistically, GP-LVMs can be considered as a form of factor analysis and related to classical approaches such as principal component analysis while adding the capability for nonlinear dimensionality reduction. A GP-LVM is described by a set of Gaussian kernels that jointly define an LV space and a set of anchor points that encode a representation of a subset of the data (or interpolations thereof) within that space. The particular variant of GP-LVM that we used in this article employs a Bayesian method to extract LVs and to optimize the set of anchor points [32,33]. An important extension of the GP-LVM approach is *manifold relevance determination* (MRD), which allows the joint optimization of two or more LV models each derived from different modalities or *views* [33–35]. MRD is predicated on the idea that each view contains information that is private or exclusive to that view, together with information that is shared across the views. The *multiview* learning capability enabled by the MRD algorithm allows the different views to decide the importance (if any) of each latent dimension to that view, while the shared view extracts LV dimensions that capture the variance that is common across views. Further extensions of the GP-LVM approach that could be useful for modelling human memory include the ability to stack LVMs in hierarchies [36] or as ‘deep’ models [37].

We will hereafter refer to the use of a single GP-LVM within our robot memory system as a *simple synthetic memory* (SSM). As described below, we have primarily applied SSMs to encode memories within a single sensory modality/stimulus type; however, SSMs can also be trained to efficiently encode memories across heterogeneous sensory modalities, using the multiview approach, or within a broader framework, to combine memories across modalities or within an event (see §4). We next describe the operation of an SSM for face recognition as an illustration of this approach.

**Figure 2.**
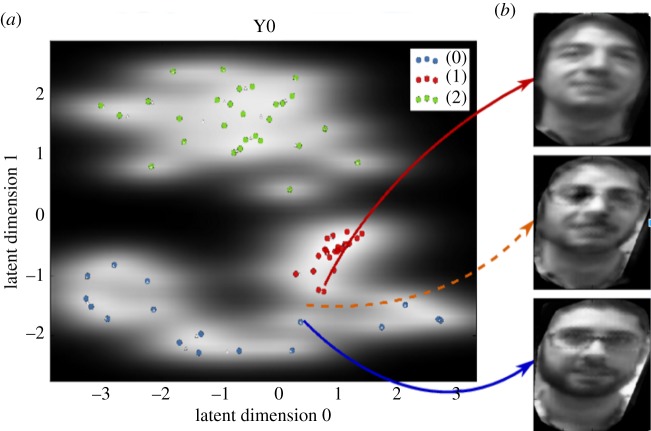
A two-dimensional SSM for faces. (*a*) The two-dimensional LV space obtained from the high-dimensional face image data; coloured points represent saved anchor points. The model was trained in an unsupervised way, without the provision of class labels; cluster of points corresponding to the three individuals used in the training set are colour-coded for display purposes. (*b*) Reconstruction of faces from three locations in the space. The orange dotted line shows the reconstruction of an imagined face that merges features from two different people. © 2016 IEEE. Reprinted, with permission, from Martinez-Hernandez *et al.* [38].

**Figure 3.**
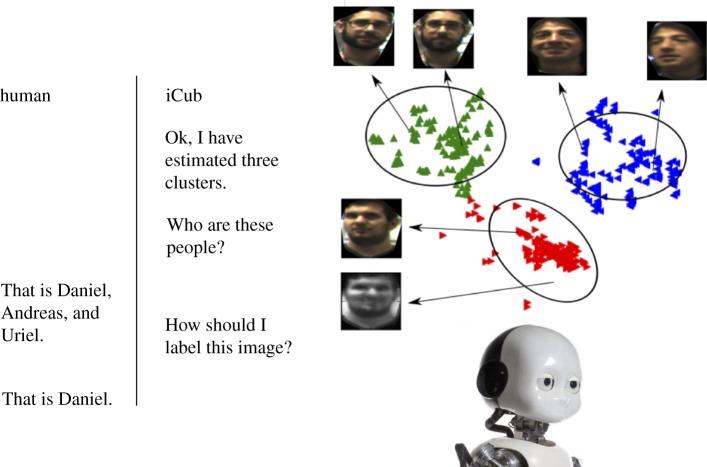
An interaction scenario for resolving uncertainty. In the example shown, iCub is provided with data corresponding to unlabelled faces; it then forms a representation of these data in the LV space and finds clusters of data points within that space using an unsupervised learning method such as automated *k*-means (http://dx.doi.org/10.14419/jacst.v4i2.4749) (Ok, I have estimated three clusters); iCub is then able to request labels (names) for each cluster sequentially (Who are these people?). In parts of the space where the system has low certainty, it can generate an image of a face and request a label for it (How should I label this image?).

Figure 2 shows a two-dimensional LV space generated using stimuli extracted from the visual feed provided by the cameras on our iCub robot and consisting of multiple images of the faces of three individuals. These faces were detected using a standard template-based face-detection approach but then extracted as rectangular 200 × 200 greyscale pixel patterns (see [35] for details). An SSM model was generated, as shown in the figure, where the two-dimensional LV space encodes patterns in the 40 000-dimensional space of face image data. In the figure, the coloured points show the locations in LV space of the observed faces and the images on the right show reconstructed faces corresponding to three locations within the space. The background grey-level pattern across the space indicates the system's estimate of how certain it is of its ability to reconstruct the sensory stimulus—dark areas indicate low certainty, and light areas high certainty. In an interaction scenario, uncertainty could be used to trigger certain types of social interaction, for instance, requesting confirmation of identity (e.g. in figure 3), or could be used to decide how much weight is given to evidence from different sensory modalities. In the model shown, the system is confident in reconstructing faces in areas near to the observed data, indicated here by the two faces labelled with the solid red and blue arrows. It is less certain in unsampled areas, such as that labelled by the dotted orange arrow, where it nevertheless reconstructs a plausible face that merges the appearance of the two nearest individuals.

Note that chunking and pattern separation are naturally manifested within the SSM formulation. For instance, when a set of faces is presented to the model, the low-dimensional encoding naturally clusters similar patterns within the latent space that typically correspond to different individuals. In the robot implementation described in §3, we represent the class labels (people's names) as a second latent space and use the multiview approach to optimize the global separability of both latent spaces.

## 3. A multimodal memory system for the iCub robot

To validate our approach, we have implemented a multimodal memory system for the iCub humanoid robot and have demonstrated its ability to learn and recognize faces, voices, actions and touch gestures through interaction with people. Further details and pointers to code and data files are provided in the electronic supplementary material; in this section, we describe the different modes of memory retrieval available in this system and explain how we have implemented SSMs for a number of different modalities/stimulus types.

### (a) System implementation

**Figure 4.**
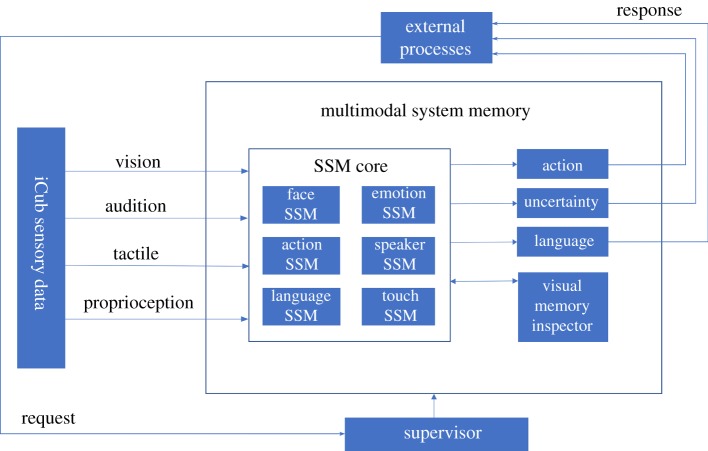
Implementation of a multimodal memory system for the iCub humanoid as a set of SSM models. The SSM core receives input from all of iCub's sensory modalities and extracts the relevant features for the different models. During human–robot interaction, a classification request can be sent to the supervisor by an external process, such as reasoning or action planning, and a response is computed and returned to the external process that requests it in the form of an action, uncertainty measure or language token (or some combination). At the same time, the visual memory inspector can provide a visualization of the items recognized by the SSM core.

Our implementation of a multimodal memory system, as illustrated in figure 4, includes the set of LV models together with the supervisory computational processes needed to query the system (classify incoming sensory data and/or to generate sensory reconstructions), and to control data acquisition, training and model optimization.

Taking the example of face recognition, the process starts with the collection of a small labelled dataset of approximately 400 images per person (this constitutes 2–3 s of interaction with the iCub with the template extraction running). In the current implementation, the supervisor recognizes the addition of a new dataset and allows the user to train relevant SSM models. Work is in progress to implement online learning/updating of the SSM models using graphical models (see §4) to obviate the need for any human intervention in the training process.

During human–robot interaction, different modes of memory retrieval are available to control processes external to the multimodal memory system that can initiate memory retrieval and potentially trigger an associated response from the robot such as an action or a verbal report. Our current implementation uses three different sampling modes—*continuous, past_buffered* and *future_buffered*—as described next. Each mode specifies how the incoming sensory data are collected, classified and used by the memory system, but all sampling modes operate using the same core SSM models.

Psychologists have distinguished two kinds of memory retrieval—*voluntary* and *involuntary* [39]. Involuntary memory is the spontaneous recovery of a memory triggered by a stimulus or context. The sensory cues that trigger involuntary memory are typically central features of the remembered event—you see a face and you are reminded of who it is and the last time you met—this helps you frame the interaction you are about to have. The first mode of operation of our system, *continuous*, constantly monitors the incoming data stream and a classification happens as soon as a previously trained percept is recognized. When this happens, the classification is stored in a buffer and a trigger event is broadcast that could be used by external processes (e.g. to greet the user). The continuous mode of operation is similar to the notion of human involuntary memory, because memory retrieval happens automatically whenever an appropriate stimulus is received.

Human voluntary memory, by contrast, is the active recall of a specific memory. In the laboratory, you might be presented with a sensory cue, such as a picture of a friend, and be asked to generate related memories. In everyday life, you might try to remember the last time you saw your friend, and this might invoke the memory of a visit to a coffee shop last Thursday and of the conversation that you had there. In the case of both *past_buffered* and *future_buffered* sampling, the robot explicitly triggers a request for a classification within an SSM. The difference between these two modes depends on whether the stimulus pattern that is used to cue recall is already held within a sensory buffer (*past_buffered*), or the robot actively seeks for a relevant stimulus in its environment (*future_buffered*); an example of the latter would be to search for a face to classify at the start of a human–robot interaction episode. The *past_buffered* version of recall can be used to explicitly interrogate the system for specific memories and therefore has similarities to human voluntary memory.

The pattern completion capabilities of SSMs mean that memories are content addressable; for instance, part of a face can be used to retrieve a whole face, or a feature such as long hair or glasses could be used to retrieve plausible matches. Note that SSMs also allow for the construction of fantasy events or patterns that are possible but have never been observed (e.g. [35]); in other words, they can implement a form of *imagination*. In this case, a label is sent to the system to identify which class of sensory data is to be generated and the supervisor samples the relevant SSM to generate an exemplar.

### (b) Simple synthetic memories for different modalities

**Figure 5.**
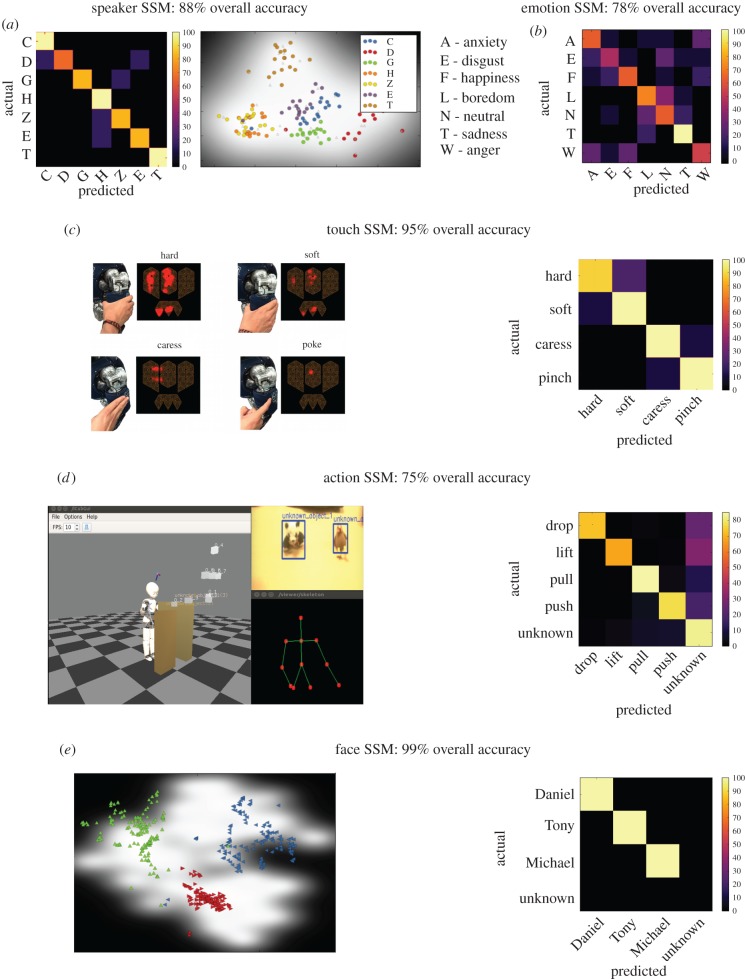
Results achieved using multiple SSMs trained for human–robot interaction. (*a*) Speaker SSM confusion matrix and LV space; letters indicate different speakers. (*b*) Emotion SSM confusion matrix. (*c*) Touch SSM example input data of contact with the iCub forearm (red areas indicate active tactels) and confusion matrix (from Martinez-Hernandez *et al.* [38]). (*d*) Action SSM example data (user stick figure and object segmentation) and confusion matrix (from Camilleri and Prescott [40]). (*e*) Face SSM confusion matrix and LV space. In the confusion matrices, correct classification is represented along the diagonal (top left to bottom right), good classification is indicated by light colours along this diagonal, and by dark colours elsewhere. Further details of the training data and results are provided in the electronic supplementary material.

**Figure 6.**
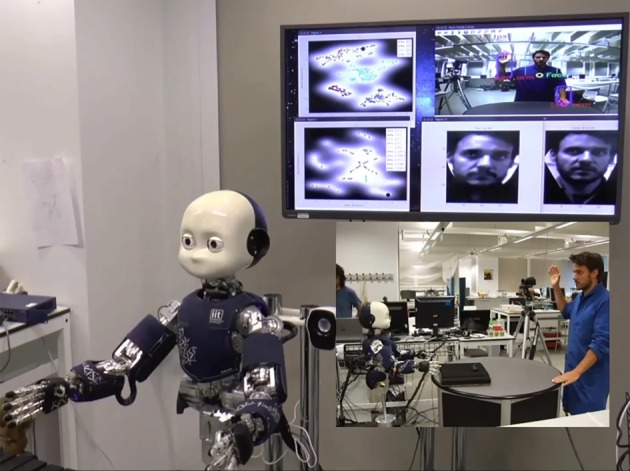
Real-time operation of iCub SSM models for faces and actions. The inset shows a side on view of the scene that includes the human interlocutor. The large video screen behind iCub shows the real-time view from iCub's right-eye camera with the face and arm/hand areas segmented, the LV spaces for faces (top left) and actions (bottom left), and the face extracted from the current scene (bottom centre) and its reconstruction (from the face SSM).

Figure 5 presents results obtained from the current implementation of iCub's multimodal memory showing five different SSM modules and confusion matrices indicating their accuracy for exemplar training/test datasets. The figure also shows acquired LV spaces for the SSMs supporting person recognition from audio and visual data streams. Figure 6 illustrates the face and action recognition SSMs operating in real time during a human–robot interaction.

We have already described some of the capabilities of our system for storing and recalling faces, next we summarize memory formation and retrieval of human voices, haptic interactions and actions performed on objects.

#### (i) Audition

Our auditory models have so far focused on human speech as a key element of human social interaction. We focus on social cues available from speech rather than on spoken word recognition; for instance, speech can be used to identify a speaker and his/her emotional state. To encode auditory memories, we use *Mel-frequency cepstrum coefficients* (MFCCs) [41] to construct data vectors for training an SSM. MFCCs are a standard approach in speech processing that characterizes the speech signal in terms of the power present in several frequency bands (and as such approximate the Fourier transform of the power spectrum of an audio frame). In the first stage of feature extraction, an utterance is extracted by segmenting the audio based on periods of silence. Subsequently, this utterance is divided into frames of equal temporal length and MFCC features are extracted from each frame.

A dataset for auditory speaker recognition was collected by asking participants to read a collection of sentences; these recordings were then segmented into utterances and the SSM trained/tested on an 80/20 split. The speaker recognition SSM, illustrated in figure 5*a*, demonstrates good clustering and separation of the different participants and also shows clustering of similar voices close to each other when taking into consideration the nationality/accents of the different participants. Specifically, C, D and G are French and thus clustered close to each other. Conversely, T is German, D is Maltese, while H is Korean and M is Italian. The emotion recognition SSM shown in figure 5*b* was trained on data from the Berlin Database of Emotional Speech [42], achieving an overall accuracy of 78% on an 80/20 split of the dataset. The confusion matrix indicates good separation of the classes other than ‘boredom’ and ‘neutral’ which are, perhaps unsurprisingly, hard to distinguish. Work is in progress to validate emotion recognition using data from real-time interactions in our own laboratory.

#### (ii) Haptic interactions

In the case of haptic interactions, we wanted to be able to identify different classes of user physical interaction with iCub's ‘skin’ during social touch. We defined a set of haptic interactions, such as hard touch, soft touch, caress and poke, and formed a dataset by asking participants to interact with the tactile surfaces on iCub's arms in these different ways. The touch SSM was able to recognize the different stimulus classes with an overall accuracy of 95% [38]. We have shown elsewhere that a tactile memory of this kind can be used to close the loop between social touch and the robot's expressed response [43,44].

#### (iii) Actions

To identify actions performed on specific objects, we combined stereo vision and depth tracking of objects using iCub's cameras with the mapping and depth tracking of a stick figure frame of the user using a Microsoft Kinect^™^ sensor (see [45] for details). A particular challenge here was the integration of different sensory sources with different time lags and sampling frequencies as well as the inherent temporal structure of the task. As detailed in Camilleri and Prescott [40], our approach used a temporal to spatial conversion that mapped periods of action into labelled intervals. We used the object location from iCub's cameras together with the hand location of the agent interacting with iCub to classify a set of four actions, performed on graspable objects within iCub's field of view. The action recognition confusion matrix, shown in figure 5*e*, demonstrates that this model is capable of correctly classifying an action 75% of the time with the remaining 25% being false negatives with no false positives.

#### (iv) Face recognition

The results for the face recognition SSM, which used a multiview approach to associate names with faces, are shown in figure 5*f*. The figure shows a well-clustered latent space that distinctly encodes three faces in two dimensions as expected from the pilot study data shown in figure 2.

#### (v) Reconstructing the sensory scene

**Figure 7.**
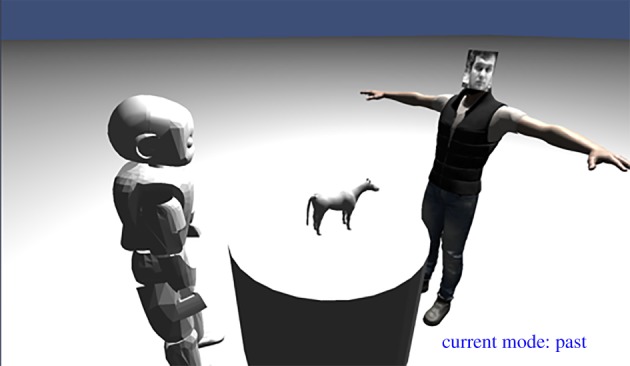
Generation of objects of interest and agents from a textual description of past memory within the VMI; recalling the scene within the context of a three-dimensional simulator that includes simulated physics could allow iCub to imagine and rehearse possible actions within the scene.

As previously noted, recall and imagination are generative processes that involve the rekindling of activity in primary sensory areas including visual cortex. To express this, we have developed the *visual memory inspector* (VMI), illustrated in figure 7, to provide a window into the ‘first person’ world of the iCub [46]. The VMI builds on a physics-based, three-dimensional simulation engine which is integrated with the multimodal memory to visually display how the current scene, or a restored memory, is represented by the iCub. This is done by taking the current classification labels for all SSM models, if we are visualizing the present, or the parsed textual description of a past memory, then using the generative capability of the memory system to re-imagine different elements of the scene. These generated recollections are then formatted into a visual description and displayed within the VMI. So far, the VMI has only been demonstrated for face recognition, but future work will extend this to actions, speech and haptic interactions. Owing to its integration with a full physics engine, the VMI can also be used to simulate/visualize actions and to plan action sequences before carrying them out in the physical world. Analogous internal simulation processes have been proposed to underlie human common-sense reasoning and action planning (e.g. [47]).

## 4. Discussion and ongoing work

The above results demonstrate that we are already making progress in our effort to provide iCub with some of the components of the memory system illustrated in figure 1; we conclude by briefly discussing how this system could be extended towards a more complete model of human autobiographical memory and MTT, and how such a system might be deployed in future social robots.

### (a) Multisensory integration and scene understanding

The Rubin model includes multisensory integration subsystems concerned with spatial and value learning. These are certainly only a subset of the multisensory representations involving human memory; indeed, purely unisensory representations in the human neocortex may be rare, even in the primary sensory areas [48]. Multisensory data can be represented in two different ways within the SSM approach. First, we can choose to consolidate multiple sensory streams within one multisensory LV space, which is the approach we took for action learning. Alternatively, and more flexibly, we can maintain different LV representations for different modalities and separately represent their shared properties; this is the multiview learning approach introduced in §2. Using this approach, the latent space of one view is mapped on to the latent space of the other and the training objective is to maximize the separation of clusters in both views while keeping an accurate, continuous and bidirectional mapping between both spaces. In the context of human memory, the multiview approach could provide a new way of thinking about multisensory activity in the cortex. For instance, studies of multisensory responses in primary sensory cortices suggest possible roles for cross-modal interactions in attention regulation, stimulus enhancement and in reducing trial-to-trial variability [49]. Thus, rather than directly representing the stimulus properties of the other modality, the coordination across two modalities could be concerned with enhancing the representation of unimodal information within each separate modality as suggested by the multiview approach.

For our robot memory system, we are interested in using multiview learning to compactly represent what is shared by sequences of camera frames while also encoding information about what is unique in each different frame. This can provide an efficient way to encode temporal sequences (see below for discussion and additional approaches). We are also developing multisensory SSMs that combine vision, touch and proprioception to create a robot self-model and to represent the peripersonal space around the robot (see [50–52] for related work in this direction). Such models could provide the basis for safer human–robot interaction, and could support the development of a robot self-other distinction [3].

Deep learning approaches have been shown to be powerful methods for recognizing complex, high-level features in sensory scenes; moreover, the different feature-types abstracted in successive layers of such networks can show interesting similarities to the gradient of complexity in the human ventral visual pathway [53]. Deep GP-LVMs could show a similar capacity to extract useful representations while learning from much smaller datasets [37], thus coming closer to our human ability to learn from a small number of examples [54]. Hierarchical GP-LVM models also show promise for efficiently encoding temporal sequences and for constructing structured representations of complex scenes [36].

### (b) The event memory subsystem

In the theoretical framework, we are investigating whether a key role is served by the event memory subsystem that binds together the different components of an event, both synchronously and sequentially, and does so in a way that allows similar but distinct events to be maintained as separate memory traces. Hasselmo [26] has provided a detailed description and review of the *extended hippocampal system* (EHS) consistent with the proposal, originally made by Marr [25], and further developed through a large number of models and studies, that the EHS provides the brain substrate for an event memory subsystem. Here, we briefly discuss the different elements of the EHS and consider how our robotic memory system could be extended to support their function.

On the input side of the EHS, the entorhinal cortex (EC) acts as the convergence zone, via parahippocampal and perirhinal cortices, for information from different sensory modalities. Investigations of EC indicate a ‘what versus where’ distinction in the types of information encoded by its antero-lateral and posterior-medial areas [55]. Responses in antero-lateral EC, to stimuli such as physical objects and human faces [56], are consistent with a low-dimensional encoding of non-spatial features of the physical and social world, and their emotional value, as might be delivered by our SSM models. The discovery of grid cells in the medial EC in rats [57], and evidence of the role of human and rat EC in temporal coding [58], implies a parallel encoding of the spatio-temporal features of events. The dentate gyrus (DG) receives divergent input from EC and provides a substrate that could support sparse encoding, thereby increasing dimensionality and improving pattern separation; this interpretation is supported by imaging data showing better separation of activity patterns generated by similar events in DG compared to either EC or CA3 [59]. The attractor dynamics of the hippocampal circuits in CA3, which receive inputs from EC and DG, is thought to support an associative memory that provides rapid binding of the different elements of an event (as discussed further below). Finally, CA1, and pathways back to EC via the subiculum, could provide decoding of CA3 patterns into stimulus representations that could be matched against incoming data to validate predictions (e.g. [60]) or reconstructed elsewhere in the brain as patterns of remembered/imagined experience.

Various mechanisms have been proposed that could underlie the storage/retrieval of sequential memories in the EHS (see [26] for review). These include recurrent connectivity in CA3, the grid cell representation in EC [57,58], ‘time’ cells in CA3 [61] and the overall attractor dynamics of the EHS (e.g. [62]). Neuronal activity could specifically encode timing within an event or could participate in generating a temporal sequence through mechanisms such as theta phase precession (e.g. [63]). Various approaches to sequence encoding have been investigated with GP-LVM models. These include converting temporal patterns into structural ones, as in our models of voices and actions discussed above, and using multiview learning to optimize storage across and within frames [33,34]. Dynamical models have also been developed that introduce prior information about the temporal nature of the encoded information (such as smoothness and periodicity) into the Gaussian kernels. This method has been successfully used to capture temporal patterns from motion capture data of people walking or running [33].

**Figure 8.**
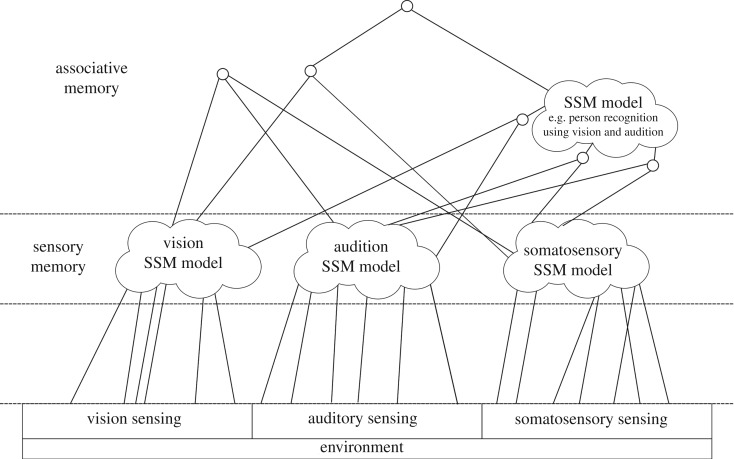
A hybrid approach to event memory. An architectural visualization of hybridizing graphical models with SSM models to address the challenge of rapid and one-shot learning for episodic/autobiographical memory. The architecture is split into three layers: (i) an input layer, (ii) a unisensory layer composed of single modality SSMs, and (iii) a multisensory associative memory represented by nodes, edges and higher-level multisensory SSM models. The graphical part of the model allows for the rapid encoding of one-off events. After the rapid encoding is carried out, these nodes are clustered with respect to the information that they contain and groups of homogeneous nodes can then be used to train SSM models.

It is a basic requirement of episodic/autobiographical memory to be able to store and retrieve an event that only happens once; however, this is a major challenge for models of the EHS based on associative memory (see [26]). The SSM approach faces the same difficulty which is the requirement to have a dataset containing multiple instances in order to configure an LV space. It is worth noting that the data requirements for an SSM are relatively low when compared with approaches such as deep neural networks; however, we still require multiple data points that are homogeneous to a given sensory modality/stimulus type. To address this challenge of rapid, one-shot learning, we are investigating the use of hybrid multilayer models that mix probabilistic graphs with SSM models to implement a form of fast associative memory (see figure 8 for an example illustration). The graphical part of the model allows for the encoding of events as they transpire (for an example of unsupervised graphical modelling of everyday human activity, see [64]); this resembles CA3 as it should be capable of rapidly encoding event information across multiple modalities. After the graphical encoding is carried out, these nodes can be clustered with respect to the information they contain and the data attached to groups of homogeneous nodes then used to train SSM models. This process, which can occur offline, would allow for the generalization of events that occur frequently, while reducing the overall size of the graph.

### (c) Memory as symbol grounding for robot social cognition

Together with colleagues, we have also been incorporating SSM memory models into a brain-inspired control architecture for the iCub robot called *distributed adaptive control* (DAC) [65,66]. This system is being advanced towards an integrated model of social cognition for human–robot interaction (see [45,52]) that includes a reactive interaction engine, goal-directed behaviour based on a simulated motivation/drive system, and state-of-the art subsystems for perception, motor control and planning. The DAC framework provides a high-level conceptual scheme that seeks to capture the cognitive architecture of the human mind and that consists of four tightly coupled control layers. Whereas the lower three layers, which provide somatic, reactive and adaptive capabilities, operate largely in the here and now, the fourth ‘contextual’ layer adds the ability to store and retrieve event memories, linked to goal achievement, that can also act as action plans. The SSM modules described here can be conceived of as belonging to the DAC adaptive layer but contributing to the construction of episodic and declarative memories at the contextual level. The contextual layer also includes the ability to form symbolic representations of events in narrative (linguistic) form that allow the robot to summarize and communicate about its past experiences. Using this narrative system, iCub can recall and discuss past events, including some of its past interactions with people, from a first-person perspective. In other words, this architecture is able to support some of the functions of the ‘narrative’ and ‘search’ subsystems in figure 1 (see [67] for further details). One longer-term goal is to integrate this narrative construction process with an SSM-based memory system such that narrative descriptions can be abstracted from representations of events as patterns in LV space via the capacity, using multiview learning, to acquire linguistic labels for people, objects and actions. Using the generative capabilities of SSMs, retrieved narratives could also be played out as simulated sensory scenes through the VMI.

We consider that linking narrative, and symbolic/linguistic processes more generally, with sensory memory via SSMs can provide a solution to the problem of ‘symbol grounding’ in artificial intelligence [68] and thus play a role in generating intentionality—the capacity of mental states to be about something. Specifically, we propose that the kinds of representations provide by SSMs match the requirements suggested by Harnad [68] for grounded representations in two ways. First, by employing learning to form non-symbolic low-dimensional encodings of the invariant features of salient perceptual categories—such as objects, people and events in the robot's environment—SSMs encode internal representations that have a non-arbitrary mapping to properties of the external world. Second, by allowing, through the generative capabilities of SSMs, the reconstruction from linguistic tokens of sensory patterns that are similar to those impinging on the robot's sensory transducers, we demonstrate that those tokens, and any symbolic manipulation that employs them, can be grounded in patterns of activity that are similar to the ‘proximal sensory projections of distal objects and events' [68, p. 335]. In other words, via a loop through the robot's memory system, sensory experience can be encoded into a linguistic form that can support forms of symbolic/narrative reasoning then decoded back into patterns of sensor activity that resemble those generated by direct experience.

### (d) Using brain-inspired memory systems in social robots

While this work is still at an early stage, we are already seeing some potential benefits of deploying memory systems modelled on those found in the human brain in social robots. For instance, integrating SSM models in the DAC architecture for iCub has enhanced the robot's capability for recognizing social actors and actions [45,52]. We foresee the following benefits from the further development of the systems we have described here towards a more complete model of human autobiographical memory and MTT:

(i) The involuntary mode of memory retrieval should assist a robot to fill out its current understanding of a social setting both by recognizing actors and their actions and by recalling aspects of past interactions with those actors, including their emotional associations. Pattern completion triggered by social cues should help the robot to retrieve information that is relevant (thus helping to circumvent the AI frame problem) and should assist the robot to select behaviour that is more appropriate to the current setting and better matched to user needs.
(ii) The voluntary mode of retrieval could be used to retrieve specific memories of past interactions on request and could serve as a reminding function. For instance, the robot could recall a past interaction and describe it to, or recreate it for (via the virtual memory inspector), the human interlocutor.
(iii) The capacity to reconstruct past episodes could serve as the basis for planning future behaviours, using memory for outcomes to decide if sequences of action are applicable and worth repeating. This is already happening in the DAC architecture where action plans, represented as graphical models, can be accessed based on goal cues extracted from human speech. The memory models we are developing should allow new plans to be abstracted from experience.
(iv) The virtual memory inspector could also be used as an action planning system to rehearse behaviours before performing them in the real world, and to internally observe whether they achieve their intended consequences.

Beyond these specific uses, we see a role for biomimetic memory systems, alongside systems for narrative construction and internal simulation, in providing robots with the ability to represent themselves as entities that exist, persist and act in time. Adding the ability to model the same capacity in others (e.g. [69]), within a broader human-like cognitive architecture [52], will lead to artificial others that are able to better comprehend, and participate in, our human social world.

## Supplementary Material

Memory and Mental Time Travel in Humans and Social Robots: Electronic Supplementary Material

## Data accessibility

The supporting data are provided in the electronic supplementary material.
