# Supplementary material for: Memory and mental time travel in humans and social robots
Source: Philos Trans R Soc Lond B Biol Sci. 2019 Mar 11;374(1771):20180025. doi: 10.1098/rstb.2018.0025 (PMC6452248; doi:10.1098/rstb.2018.0025)
Supplement: Memory and Mental Time Travel in Humans and Social Robots: Electronic Supplementary Material [file rstb20180025supp1.docx]

^Memory and Mental Time Travel in Humans and Social Robots:^

^Electronic Supplementary Material^

### Training data and results

| SSM | Total Number of Samples | Data Classes | Useability | Training accuracy | Testing Accuracy |
| --- | --- | --- | --- | --- | --- |
| Speaker | 15 mins of speech | 7 | 100% | 93% | 88% |
| Emotion | 25mins of speech | 7 | 100% | 88% | 78% |
| Touch | 150 touch events | 4 | 100% | 99% | 95% |
| Action | 20,800 data frames | 8 | 26% | 100% | 75% |
| Face | 1020 images | 3 | 80% | 100% | 99% |

* Useability describes the percentage of the total data that can be partitioned into the data classes. Training accuracy is measured on a fraction of the useable data only. Testing accuracy is measured on all samples.

### Accessing Data and Code

Please follow the instructions at <https://github.com/dcam0050/docker_starter> contained within the README. Follow the section to set up docker and then the SSM specific section for instructions on how to prepare and use the installation.

For additional information on how to use SSM please refer to <https://github.com/dcam0050/ssm>
